# Medical and Physical Intelligence

**Published:** 1803-10-01

**Authors:** 


### MEDICAL AND PHYSICAL INTELLIGENCE.

[ FOREIGN AND DOMESTIC. ]

*Extract of a letter from the Baron Delaferronnays to the Editors, dated Weymouth Street, Sept.* 12, 1803.

" A few weeks ago the 51st Number of the Medical Journal came into my hands; and in your Address to Correspondents, I observed that you had received several accounts of the success of the Iceland Lichen in pectoral complaints. In this article it is observed, that if the remedy were more completely employed, and, above all, that if practitioners and their patients had more perseverance in the use of it, instances of its success would be far more frequent. Permit me to support this opinion by relating the case of Madam the Baroness Delaferronnays, my lady, who owes her present health entirely to the steady use of this lichen both as medicine and aliment.

" This lady had laboured under a humid asthma for several years, which proved extremely distressing to her, as she was often nearly suffocated; and whenever she attempted to walk more than 200 paces, the stricture across the chest obliged her to rest. A cough, not less violent than frequent, tormented her continually, which was attended with an abundant expectoration, streaked with blood, and of a purulent appearance. The fever, loss of appetite, and even of sleep, alarmed me in the greatest degree. Hearing of Dr. Regnault's success in treating diseases of the chest, I consulted him about eleven months ago; he recommended the Baroness to take the decoction of the lichen with the concentrated syrup, and to breakfast every morning on the Iceland chocolate,* with a suitable diet. In about fifteen days she appeared to me evidently better; the fever had abated, and the expectoration seemed of a better

---

* These preparations are explained in Dr. R's book, and may be had at Mr. Huguenin's, No. 33, Haymarket. Ed.

ter colour; her appetite and sleep returned; and by the uninterrupted use of the lichen and its chocolate jointly, under the care of Dr. R. the shortness of breath on walking, and the suffocating attacks which had so long harrassed the Baroness, entirely left her; and it is now four months since she left off the use of a remedy, for which she had no farther occasion. I judged it right, as well for the cause of humanity as that of truth, to send you this account, being persuaded that these are the objects of your Journal.

"Case II. Mrs. Swinburn, No. 12, Great Quebec Street, has been, since last winter, subject to harrassing defluxions of the head. About the month of May, after a severe affliction, she was seized with a violent spasmodic cough, followed by an inflammation of the lungs. All the usual means were employed, such as venæsection, blisters, &c. but the fever, which had been acutely inflammatory, assumed the character of a low fever, attended with a continual cough, expectoration of a purulent appearance, and extreme weakness. This patient, resigned to death, had settled her spiritual and temporal affairs, when, in the month of July, she commenced the use of the Lichen Islandicus. Its effect has been astonishing; the cough diminished, the expectoration became easy, her strength returned, the fever disappeared, and at the end of a month Mrs. S. was perfectly cured. She continued the use of the lichen, however, some days longer, and at the end of August she left it off, finding her health better than it had been for many months before."

---

*Extract of a Letter from our Correspondent at Paris, dated August*, 1803.

"Paris has for some days rung with relations of the wonderful exploits of a Spaniard in this city, who is endowed with qualities by which he resists the action of very high degrees of heat, as well as the influence of the strong chemical re-agents. Many histories of the trials to which he has been submitted before a commission of the Institute and Medical School, have appeared in the public papers; but the public wait with impatience for the report to be made in the name of the commission by Professor Pinel.

"Until this report, which will contain a variety of details on the mode of conducting the experiments, be made known, your Correspondent sends some of the more remarkable circumstances, of which he has been himself a witness.

"The subject of these trials is a young man, a native of Toledo in Spain, twenty-three years of age, and free of any apparent peculiarities which can announce any thing remarkable in the organization of the skin; after examination, one would be rather disposed to conclude a peculiar softness than that any hardness or thickness of the cuticle existed, either naturally or from mechanical causes. Nor was there any circumstance to indicate that the person had been previously rubbed with any matter capable of resisting the operation of the agents with which he was brought in contact.

"This

"This man bathed for the space of six minutes, and without any injury either to his sensibility or the surface of the skin, his legs in oil, heated at 97 deg. of Reaumur, (250¼ deg. of Fahrenheit*); and with the same oil, at the same degree of heat, he washed his face and superior extremities. He held for the same space of time, and with as little inconvenience, his legs in a solution of muriate of soda, heated to 102 of the same scale, (261½ Fahr.) He stood on and rubbed the soles of his feet with a bar of iron heated to a white heat; in this state he held the iron in his hands, and rubbed the surface of his tongue.

"He gargled his mouth with concentrated sulphuric and nitric acids, without the smallest injury or discolouration; the nitrous acid changed the cuticle to a yellow colour; with the acids in this state, he rubbed his hands and arms. All these experiments were continued long enough to prove their inefficiency to produce any impression. It is said, on unquestionable authority, that he remained a considerable time in an oven heated to 65 or 70 degrees (178—189 Fahr.) and from which he was with difficulty induced to retire, so comfortable did he feel that high temperature.

"It may be proper to remark, that this man seems totally uninfluenced by any motive to mislead, and, it is said, he has refused flattering offers from some religious sectaries of turning to emolument his singular qualities; yet, on the whole, it seems to be the opinion of most philosophical men, that this person must possess some matter which counteracts the operation of these agents. To suppose that Nature has organized him differently, would be unphilosophic; by habit he might have blunted his sensibility against those impressions that create pain under ordinary circumstances; but how to explain the power by which he resists the action of those agents which are known to have the strongest affinity for animal matter, is a circumstance difficult to comprehend. It has not failed however to excite the wonder of the ignorant, and the inquiry of the learned at Paris.

---

*Observations on the Barbary Coast; communicated to* Dr. MITCHILL, *by* Dr. GEORGE DAVIS, *Surgeon to the American Fleet.*

I have been in Tangiers, but can offer few remarks on the place. The plague prevailed in this town, a few years since, with much mortality. If it proves epidemic in Tangiers, with all the advantages of its local situation, its commercial intercourse with the adjacent countries, as also its being the residence of all consuls, who ought to be supposed to have some influence with the police of the country, particularly as it relates to the general health; the other dominions

---

* As the method of converting the degrees on Reaumur's thermometer to those on Fahrenheit is not generally known, we insert the rule: multiply the number on Reaumur by 2¼, and add 32 to the product. The heat of boiling water is 212° of Fahrenheit.

dominions of the Emperor of Morocco will always be harrassed, in a greater or less degree, by this child of filth and nastiness.

The lower class, who are indeed three-fourths of the inhabitants, contrary to the injunctions of Mahomet, neglect, in a most beastly and filthy manner, their persons, as it respects cleanliness; a woollen waistcoat, with a haick, or a coarse woollen frock belted round, being their only dress, and which, I am told, is often worn without shifting, until the ravages of time save them the trouble of removing it. The mode of building their houses, which are low, without any windows, and communicating one to the other from the roof, must have its influence.

The streets are narrow throughout all Barbary, and the receptacle of every species of filth and nastiness. The extreme poverty and indolence of the people, together with the miserable diet on which they subsist, must prove powerful agents in the production of this disease, which, once set in action, is greatly assisted by what they conceive the only means of arresting its progress.

---

Dr. Trotter, in a letter to the Editors, dated Newcastle upon Tyne, August 14, 1803, says:

"About ten years ago, I had it in contemplation to comment on the subject of my Inaugural Dissertation;* and was forming the plan of an Essay on Drunkeness, and its Effects on the Human Body, when I was ordered to sea, at the beginning of the late war. To this task I was recommended by my friend and preceptor Dr. Cullen, and the present Professor of the Practice of Medicine in Edinburgh. Studies of a different nature having from that time engrossed my attention, it was not till now that I was able to resume my inquiry.

To such of your Readers, who have had any considerable share of the practical part of our art, or have profited by an extensive intercourse with mankind, this subject will appear of great importance. It is certainly one, as connected with the duties of our profession, that requires more address and knowledge of human nature, than almost any other. The phenomena attendant on ebriety, draw forth some of the most singular and interesting propensities that are to be met with in the character of rational beings. The life of a fellow creature often depends on the treatment of the drunken paroxysm; frequent intoxication paves the way to a multitude of diseases; it exhibits in glowing colours the strong attachments of habit, and how difficult to be overcome; in short, independant of the moral evils which it occasions, no human infirmity stands more in need of medical investigation.

Should

* Dissertatio Medica

Inauguralis

Quædam, De Ebrietate

Ejusque Effectibus

In Corpus Humanum

Complectens.

Should, therefore, any of your Readers be disposed to favour me with any Facts, Cases, Histories, Cures, or Dissections that relate to my subject, I shall most gratefully receive them, and most faithfully acknowledge their communications.

---

Mr. Carpue, in the presence of Dr. Pearson, and several other medical gentlemen, has repeated the Galvanic experiments on the body of Michael Carney, the criminal lately executed in London for murder. The principal object was to ascertain, whether Galvanism, applied immediately to the nerves, could excite action in internal parts, and particularly those subservient to respiration. With this view, an opening was first made into the wind-pipe, and about three pints of oxygen gas thrown into the lungs; the phrenic nerve was then exposed to conductors applied to it, and the inside of the rectum, the lungs being at the same time occasionally inflated; yet no action could be excited in the diaphragm;—the nerves do not seem to be conductors of the Galvanic fluid. Conductors applied to the inside of the rectum and nostrils, excited very considerable contractions in the right auricle more than three hours after death; the ventricles were, as in Prof. Aldini's experiments, perfectly motionless; the distortions of the muscles of the countenance, &c. were nearly the same as on the former occasion. The experiments were conducted with perfect accuracy and science, but no new fact appears to be ascertained.

---

The Academy of Gottingen, which, say the foreign journals, has always distinguished itself by the importance and wisdom of the questions which it holds out to candidates, had proposed one relative to that terrible and fatal epidemic malady, called the Yellow Fever. Dr. Guttfeldt, who obtained the accessit from the Academy, has recently translated his Latin Memoir on this subject into German. He seems to think that the malady should not take its denomination from one of its accidental symptoms; it is, says he, the typhus of the tropical regions, the first notice of which we owe to Father Labat, who treated of it under the name of the *Siam Fever*, and was himself infected with it at two different periods, in the years 1694 and 1697. No further notice has been taken of it since; but during the last ten or twelve years we are unhappily but too well acquainted with its effects, though not with the real nature of the disorder itself, from the want of enlightened physicians and accurate observers. In the last century this epidemic ravaged America more than twenty times; the yellow colour is not a symptom inseparable from the malady; it sometimes does not appear even at the point of death; a blackish-hue has been found in some individuals. According to the author, the cause of this malady is a suppressed irritation, an asthenia, and the remedies proper to be applied ought to be antiasthenic. Hitherto, he observes, very few patients have recovered from it. M. Guttfeldt thinks that the decrease of bodily strength has greatly contributed to propagate the malady, which originally was not perhaps contagious.

Westminster Hospital, Sept. 22, 1803.

Mr. Lynn and Mr. Carlisle purpose, during the ensuing winter, to give Demonstrations illustrative of all the Operations in Surgery; and Mr. Carlisle will occasionally deliver Lectures on several subjects connected with the education of Surgical Pupils. These instructions will be confined to the Pupils of the Westminster Hospital only. For the Terms of the Hospital, and all other particulars, applications are to be made to Mr. Lynn, in Parliament Street, to Mr. Carlisle, in Soho Square, or at the Hospital, in James Street, Westminster, on Wednesdays or Saturdays, at half past 12 o'clock.—Mr. Carlisle will deliver an Introductory Discourse explanatory of this Plan, on Thursday, October the 6th, at eleven o'clock in the forenoon, in the Theatre of the Hospital.

---

Dr. Badham, one of the Physicians to the Westminster Dispensary, Gerrard Street, proposes to deliver during the winter, two Courses of Lectures on Chemistry.

---

John Pearson, F. R. S. Senior Surgeon of the Lock Hospital, and Asylum, and of the Public Dispensary, will commence the Autumn Course of Lectures on the Principles and Practice of Surgery, on Monday, October 3, at seven in the evening.

---

Mr. Thomas's Lectures on the Operations of Surgery, will commence in Leicester Square, about the end of October. A Prospectus may be had at his house, or at the Anatomical Theatre in Windmill Street.

---

In the course of this month Mr. Macartney will commence his Lectures at St. Bartholomew's Hospital, upon Comparative Anatomy, and the Laws of Organic Existence.

---

Mr. Moor, surgeon dentist to Her Royal Highness the Duchess of York, will commence a Course of Lectures on the Structure and Diseases of the Teeth, on the 4th of November, in which will be explained the Complete Practice of the Dentist. Further particulars may be known by applying at his house, No. 6, Palsgrave Place, Temple.

---

Dr. Blackburn's Observations on the Prevention and Cure of Scarlet Fever, with Remarks on Acute Contagions in general, are in the Press, and will be published in a few days.

